# Utilization of Nonpneumatic Antishock Garment and Associated Factors among Obstetric Care Providers in Public Hospitals of Sidama Region, Hawassa, Ethiopia, 2022

**DOI:** 10.1155/2023/6129903

**Published:** 2023-01-12

**Authors:** Merkin Bekele, Rekiku Fikre, Yitateku Alelign, Teketel Ermias Geltore

**Affiliations:** ^1^Department of Midwifery, College of Medicine and Health Science, Wachemo University, Ethiopia; ^2^Department of Midwifery College of Medicine and Health Science, Hawassa University, Ethiopia

## Abstract

**Background:**

Nonpneumatic antishock garment is one of the newly emerging technology advances that reduce blood loss which is caused by obstetric hemorrhage and help women survive during delays to get definitive care. Over 80% of maternal mortality due to hemorrhagic shock may have been prevented if a nonpneumatic antishock garment had been utilized by an obstetric care provider. However, to the current knowledge, the utilization of nonpneumatic antishock garments is low and even no single study was conducted in the study area. Hence, we found that it is necessary to assess the magnitude and factors affecting the utilization of antishock garments among obstetric care providers in public hospitals of the Sidama region, Ethiopia, 2022.

**Methods:**

A facility-based cross-sectional study design was employed among 403 obstetric care providers from June 15 to July 15, 2022. A two-stage sampling technique was applied; the data was collected by 5 trained BSc midwives using pretested and structured self-administered questionnaires. Data was entered into EpiData Manager version 4.6 and exported to Statistical Package for Social Sciences (SPSS) version 26 software. Bivariate and multivariable logistic regression analyses were used.

**Results:**

A total of 394 (97.8%) health professionals participated in this study. Overall, 30.71% (95% CI: 26.4%, 35%) of the obstetric care providers had utilized nonpneumatic antishock garments for the management of postpartum hemorrhage. Training on the antishock garment (AOR = 4.183, 95% CI: 2.167, 8.075, *p* < 0.00), tertiary hospital (AOR = 0.355, 95% CI: 0.132, 0.952, *p* < 0.04), having protocol in the facility (AOR = 2.758, 95% CI: 1.269, 5.996), availability of NASG in the facility (AOR = 4.6, 95% CI: 1.603, 13.24), good knowledge (AOR = 2.506, 95% CI: 1.26, 4.984), and positive attitude (AOR = 2.381, 95% CI: 1.189, 4.766) were significantly associated factors. *Conclusion and Recommendation*. We found that less than one-third of the study participants have used the antishock garment in the management of postpartum hemorrhage in the current study. In addition to enhancing in-service and ongoing professional development training, it is preferable to insure the availability and accessibility of antishock in the facilities in order to close the knowledge and attitude gap among obstetric care providers.

## 1. Introduction

A nonpneumatic antishock garment (NASG) is a first aid device that saves the lives of women who are in hypovolemic shock due to any type of obstetric hemorrhage [1]. As one of the most recent technological developments, NASG helps women survive while waiting for definitive therapy by treating hypovolemic shock brought on by hemorrhage and reducing blood loss [2].

The International Federation of Gynecology and Obstetrics (FIGO) recommended utilizing NASG for obstetric hemorrhage as the first aid before any other intervention or to reverse shock when other ways to control bleeding are unsuccessful [3]. Moreover, UNICEF, UNFPA, and WHO all suggest NASG as an immediate solution until women can obtain competent care [4]. The Ethiopian Federal Minister of Health added NASG to the standard treatment protocol for PPH and hypovolemic shock under its 2020 management protocols [5]. The Ethiopian Public Health Institute Public Health Emergency Management Center (PHEM) has recommended nonpneumatic antishock garment (NASG) use and affordability in all hospitals and health centers nationally [6].

The NASG is particularly applicable for use in developing nations due to its simplicity of use and low cost. In cost-effectiveness analyses, NASG has shown to be cost-effective [7]. In Hong Kong, the device costs USD 57.50, and it may be used at least 72 times [3].

Globally, in 2017, over 810 women died every day from pregnancy and childbirth-related causes, with 94 percent of all maternal deaths occurring in developing low- and middle-income countries [4]. Obstetric hemorrhage (OH), which causes around 27.1% of all maternal deaths each year, is the leading cause of death worldwide [8]. Postpartum hemorrhage (PPH) is the most common type of obstetric hemorrhage, accounting for the majority of the 14 million cases reported each year, and 25–28% of maternal mortalities are attributed due to postpartum hemorrhage [9]. Ethiopia has achieved success in reducing maternal mortality in recent decades but still has very high maternal mortality rates: 412 deaths per 100,000 live births [10]. According to the National Maternal Death Surveillance and Response (MDSR) report of Ethiopia, obstetric hemorrhage was the leading cause of reported maternal deaths, accounting for 41.3% of all deaths, and 76% of these deaths were from PPH [6]. In the Southern Nations, Nationalities, and Peoples' Region's Performance Monitoring and Accountability (PMA) report, 38 percent of all women experienced one or more complications during labor or delivery, with severe bleeding being the most common [11].

Using NASG effectively decreased maternal death by 55%, blood loss by 80%, emergency hysterectomy by 4%, and severe significant complications from 12.8% to 4.1 percent, as shown in a global study on maternal survival following PPH at tertiary-level hospitals [12]. As per CHAI data, maternal mortality due to hemorrhagic shock decreased by 80% due to the utilization of NASG between 2013 and 2015 [13].

Unless a woman receives fast and appropriate medical attention, a woman suffering from PPH can die within two hours. Mortality due to PPH is directly related to the duration and amount of bleeding [14]. Women are dying as a result of preventable causes associated with pregnancy and childbirth because the diagnosis of issues and the choice to take a woman to a health facility are frequently delayed and transportation may not be available [15].

Several policy initiatives have been introduced in many developing countries over the past few decades to improve maternal health, but these have not changed the rapidly increasing maternal mortality effectively, and the health system remains weak [16]. The UN Commission on Life-Saving Commodities for Women and Children (UNCoLSC), the Clinton Health Access Initiative (CHAI), the Safe Motherhood Program at the University of California, San Francisco (UCSF), and the Blue Fuzion Group agreed in 2015 to reduce the NASG's costs by 75% to accelerate its scale-up [17].

According to the study done at Jimma zone, 63.8% of health professionals never applied the first aid tool, NASG, for the management of PPH [18]. Ethiopia has the most rapid scale-up of NASG, followed by Nigeria, India, and Zimbabwe, but still, the utilization is low [19]. It has shown a wide range of variation from place to place in Ethiopia, with the highest rate (64.2%) in the northern Ethiopian Tigray region and the lowest in the Jimma zone with a rate of 36.2% [18].

Although there is a low utilization rate of nonpneumatic antishock garments, a few factors including sociodemographic characteristics, facility characteristics, provider knowledge, and attitude have been identified by previous studies [18, 20, 21]. Another significant worry was that even though obstetric care providers knew that NASG was available at their facility, they have not even seen or touched it [22]. It was found necessary to assess the magnitude and identify additional factors responsible for the nonutilization of NASG since the majority of previous studies included only midwives, and in fact, no single study was conducted in the study area to the best of current knowledge. Therefore, this study is aimed at assessing the utilization and associated factors of NASG among obstetric care providers in public hospitals in the Sidama region, Ethiopia, 2022.

## 2. Methods and Materials

### 2.1. Study Setting and Period

This study was conducted in selected public hospitals of the Sidama region from June 15, 2022, to July 15, 2022. Sidama National Regional State is one of the 11 regions found in Ethiopia and is located 273 kilometers from Addis Ababa, the capital of the country. It is located in the southern part of the country, and Hawassa city is the administrative city of the region. The region is bordered in the north and east by the Oromia region and in the south and west by Oromia and south regions. According to the 2007 Ethiopian Central statistical agency report estimation, the total population of the region was 4,369,214 [23]. According to the unpublished regional health bureau report of 2021, the region has a tertiary hospital, 5 general hospitals, 14 primary hospitals, 137 health centers, and 553 health posts.

Regarding human resources for health reports, 712 obstetric care providers are working in public hospitals in the Sidama region and 435 of them are midwives.

### 2.2. Study Design

A facility-based cross-sectional study design was employed.

### 2.3. Source and Study Population

#### 2.3.1. Source Population

The source population was all obstetric care providers working at public hospitals in the Sidama region.

#### 2.3.2. Study Population

The study population was all randomly selected obstetric care providers working at selected public hospitals in the Sidama region.

### 2.4. Inclusion and Exclusion Criteria

#### 2.4.1. Inclusion Criteria

Inclusion criteria include all obstetric care providers (general practitioners, nurses, midwives, IEOS, and obstetricians) working in the maternity unit of selected hospitals during the time of data collection.

#### 2.4.2. Exclusion Criteria

Exclusion criteria include obstetric care providers on annual and maternity leave at the time of data collection.

### 2.5. Sample Size Calculation

The sample size was calculated using a single population proportion formula:

(1) $$\begin{array}{c} n=\frac{{Z_{\alpha /2}}^{2}p\left(1-p\right)}{d^{2}}, \end{array}$$

where *n* is the minimum sample size required, *Z* is 1.96 for the 95% confidence interval, *P* is from the study conducted in southern zones which on utilization of NASG was found to be 48.5% [21], *q* = 1 − *p*, *d* is the margin of error, and *n* = (1.96)^2^∗(0.485∗1 − 0.485)/(0.05)^2^ = 383.8 ≈ 384.

Then by adding a 5% nonresponse rate, the total sample size was *n* = 403.

### 2.6. Sampling Technique and Procedures

A two-stage sampling technique was used. In the first stage, the simple random sampling method was applied to select hospitals. In the Sidama region, there are twenty hospitals in general (14 primary hospitals, 5 general hospitals, and 1 tertiary hospital). Forty percent of hospitals were selected by simple random sampling to get the maximum sample size, and since Hawassa University's comprehensive specialized hospital is the only tertiary hospital in the region, it was selected purposely. According to this, 1 tertiary hospital, 2 general hospitals (Adare and Yirgalem), and 6 primary hospitals (Yirba, Dore, Aleta Wondo, Wondo Genet, Hula, and Yaye) were included in this study. Based on proportional allocation to size, these 403 study subjects were distributed to each facility.

**Figure 1:**
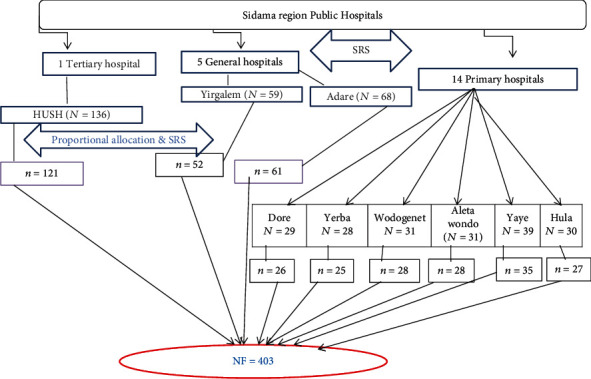
Schematic diagram of the sampling procedure for obstetric care providers of Sidama regional state public hospitals.

The total number of obstetric care providers in the 9 selected public hospitals was 451, and the total sample size was used to allocate to each public hospital proportionally. Finally, each study's participants were selected from the facility by a simple random sampling technique using a lottery method as shown in Figure 1.

### 2.7. Study Variables

#### 2.7.1. Dependent Variable

The dependent variable is the utilization of NASG.

#### 2.7.2. Independent Variable

The independent variables are sociodemographic data, health system factors, good knowledge of NASG, and positive attitude towards NASG.

### 2.8. Operational Definitions

#### 2.8.1. Utilization of Nonpneumatic Antishock Garment

Utilization of the nonpneumatic antishock garment is measured based on the response to the question of whether the obstetric care provider used NASG for the management of postpartum hemorrhage at least one time [18, 21, 24].

#### 2.8.2. Knowledge Scale

A total of 10 questions (questions 3, 4, 5, 6, 7, 8, 9, 10, 11, and 12) adapted from previous studies were used to assess the respondents' knowledge. The possible total knowledge score ranged from 0 to 27, and respondents who scored 50% and above (score ≥ 13.5) on 10 knowledge-related questions were graded as having good knowledge [18].

#### 2.8.3. Attitude Scale

The attitude was considered “positive” if the percentage score was 50% and above and “negative” if less than 50% [18, 21].

#### 2.8.4. Experienced Human Power at the Facility

Experienced human power at the facility means having a perception of the presence of healthcare providers trained on NASG in the respective healthcare facility [25].

A self-administered structured questionnaire was used to collect data from study participants. The questionnaire was developed from literature and guidelines considering the study area and situation [18, 20, 21, 25].

The questionnaire was prepared in the English version and contains five parts which include sociodemographic characteristics, facility characteristics, knowledge of nonpneumatic antishock garments, and attitude regarding nonpneumatic antishock garments and respondents' utilization of NASG.

For most of the knowledge items, participants were asked to choose the correct answer based on the nature of the items, and for some questions asked, the response was yes, no, or I do not know.

10 items were used to assess the knowledge of obstetric care providers and to calculate the knowledge score; 1 and 0 points were given to correct and incorrect answers, respectively.

The responses to attitude-related questions consist of five Likert scales: strongly disagree, disagree, neutral, agree, and strongly agree to assess attitude; 1 to 5 points were given.

The attitude statements of five Likert scales for analysis become three Likert scales; for negative statements, responses including agree and strongly agree were labeled as “disagree,” disagree and strongly disagree were labeled as “agree,” and undecided responses were labeled as “neutral responses.” Five BSc midwives having experience in data collection have been recruited and trained in data collection. In addition, the 1 MSc supervisor supervised all activities in the data collection.

### 2.9. Data Quality Control

Data collectors have provided a daylong training on the data collection tool, and each section of the questionnaire was provided to ensure that both data collectors and supervisors had the correct understanding of the data collection procedure.

Before the actual data collection, the questionnaire was pretested on 5% [20] of the obstetric care providers in the southern region, Durame town public hospital. Based on the findings from the pretest, some ambiguous questions and logical order were corrected. An ongoing formative checkup for completeness and consistency of responses was made by the supervisors daily. Data cleaning and adjustments were conducted to avoid errors in the labeling or order of the variables of interest.

Overall standardized Cronbach's alpha for internal consistency or the reliability score of attitude measurement was 0.83.

### 2.10. Data Processing and Analysis

After data collection, data were entered into EpiData Manager version 4.6 and exported to Statistical Package for Social Sciences (SPSS) version 26 software for analysis. The outcome variable was utilization; those who utilized NASG were coded as “1,” and those who did not utilize were coded as “0.” In the binary logistic regression, both bivariable and multivariable analyses were carried out. The assumption for binary logistic regression was checked. The Hosmer-Lemeshow statistic was done for model fitness and was not significant (*p* = 0.698), and the Omnibus test was significant (*p* value = 0.000) which indicates that the model was fitted. Variables with a *p* value < 0.25 in the crude analysis were the candidate for multivariable logistic regression, and those with a *p* value < 0.05 in the multivariable analysis were considered having a statistically significant association.

Multicollinearity was checked to see the linear correlation among the independent variables by using the variance inflation factor and tolerance (*t*). The variance inflation factor (VIF) for all variables was less than 1.6, and no variable has tolerance (*T*) < 0.6. The adjusted odds ratio along with 95% CI was estimated to identify factors affecting health professionals' utilization of NASG. Then, simple frequencies, summary measures, tables, and figures were used to present the information.

## 3. Results

### 3.1. Sociodemographic Characteristics of the Study Participants

Of the total of 403 eligible participants, 394 participated in this study, with a response rate of 97.8%. The mean age and standard deviation of study participants were 28.44 ± SD 3.737 years. Among the respondents, 232 (58.9%) were male. Nearly 279 (70.5%) of respondents were married. More than half of the respondents, 219 (55.6%), were protestant by religion. Professionally, 346 (87.8%) were midwives. In terms of educational attainment, 281 (71.3%) of respondents had a bachelor's degree and 273 (69.1%) had 1–5 years of clinical work experience as shown in Table 1.

### 3.2. Facility-Related Factors of Obstetric Care Providers in Public Hospitals of Sidama Region, Hawassa, Ethiopia, 2022

Regarding the distribution of participants by their organization, out of the total respondents, 162 (42.0%) of the obstetric care providers were working in primary hospitals. Of the total obstetric care providers, 86 (21.8%) had trained about NASG and 214 (54.3%) of the obstetric care providers had NASG available in their respective facilities, but from these, 105 (76.1%) know the placement of NASG after usage (see Table 2).

### 3.3. Respondents' Knowledge of Nonpneumatic Antishock Garment

Out of 394 respondents who filled out the questionnaire, 220 (55.8%) obstetric care providers had good knowledge of nonpneumatic antishock garments. This study indicated that 216 (54.6%) of the respondents correctly mentioned that NASG has six parts. Regarding contraindication to the use of NASG, nearly one-third, 253 (72.90%), of the respondents correctly mentioned that NASG should not be applied in the case of a viable fetus. About 136 (64.8%) said that nonpneumatic antishock garments are removed from women when women are awake and stable as shown in Table 3.

### 3.4. Attitude of Respondents towards NASG

The attitude of the respondents towards the utilization of antishock garments in the management of PPH as presented in Table 4 shows that 197 (50.5%) of the respondents agree that all health professionals should know about NASG. Nearly one-third of 272 (70.5%) disagree that the patient does not have to have the nonpneumatic antishock garment (NASG) applied upon. About half of the respondents, 205 (52.0%), agree that the garment should be a must in every healthcare facility that has maternity service. About 171 (43.4%) of respondents agree with the statement saying antishock garment (NASG) is only beneficial to people in rural areas/primary care settings whereas 195 (49.5%) disagree with it. Regarding total attitude, 228 (57.9%) obstetric care providers had a positive attitude towards NASG utilization.

### 3.5. Utilization of Nonpneumatic Antishock Garment

Regarding the utilization of NASG by obstetric care providers, 30.71% (95% CI: 27%, 36%) of obstetric care providers used NASG for postpartum hemorrhage management. Among those who used NASG, 46 (38.0%) of them used it during the last six months. Of the reasons reported by respondents who did not use nonpneumatic antishock garments, more than half, 228 (82.6%), were due to lack of training as shown in Table 5.

**Figure 2:**
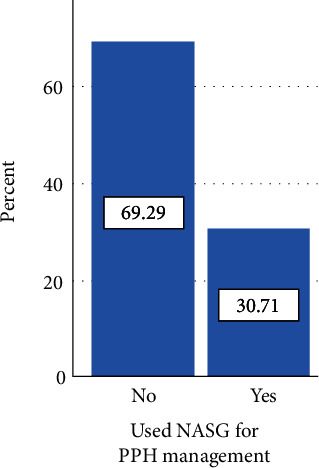
Utilization and nonutilization of NASG among obstetric care providers in public hospitals of Sidama region, Hawassa, Ethiopia, 2022.

Figure 2 shows utilization and nonutilization of NASG among obstetric care providers.

### 3.6. Factors Associated with Respondents' Utilization of NASG in Managing PPH

Before proceeding with the regression analysis, the assumptions of logistic regression and model fitness were checked using different methods. A total of fourteen variables were used for crude analysis: four sociodemographic variables (gender, job experience, professional category, and educational level), eight personal and facility-related variables (level of hospital, training, availability of NASG, number of NASG, having experienced staff, protocols in the facility, place of NASG after usage, and wall charts), and the respondent's knowledge and attitude towards NASG analyzed for associations with the utilization of NASG using binary logistic regression analysis. But there is no association between professional categories, number of NASGs, and place of NASG after usage with the dependent variable.

Six of the factors, level of the hospital, training about NASG, availability of NASG, availability of protocol, respondents' attitude, and knowledge, were found to be significantly associated with the use of antishock garments after controlling for confounders in multivariable analysis.

In this study, obstetric care providers working in tertiary hospitals are 35% less likely to utilize NASG for the management of PPH (AOR = 0.355, 95% CI: 0.132, 0.952, *p* < 0.04). Obstetric care providers who had training on NASG were 4 times more likely to use NASG as compared to those who had no training (AOR = 4.183, 95% CI: 2.167, 8.075, *p* < 0.000). The odds of the utilization of NASG among obstetric care providers who have NASG in their facility were 4.6 times more likely to use the NASG compared with those who have no NASG in their facility (AOR = 4.603, 95% CI: 1.603, 13.24, *p* < 0.005). Similarly, the obstetric care provider who has NASG protocol in their facility utilized NASG 2.7 times more likely than those who have no NASG in their facility (AOR = 2.758, 95% CI: 1.269, 5.996, *p* < 0.01).

In this study, having good knowledge has a statistically significant relationship with the utilization of NASG, and those who had good knowledge about NASG were 2.5 times more likely to use NASG (AOR = 2.506, 95% CI: 01.26, 4.984, *p* < 0.009), and similarly, those who had a positive attitude towards NASG were 2.3 times more likely to utilize NASG than those who have a negative attitude (AOR = 2.381, 95% CI: 1.189, 4.766, *p* < 0.015) (see Table 6).

## 4. Discussion

This study examined the magnitude of nonpneumatic antishock utilization among obstetric care providers working in public hospitals in Ethiopia's Sidama regional state in 2022. On the utilization of NASG, this study revealed that although 95.9% of the respondents reported they knew that NASG is used to prevent the complication of PPH, only 30.71% have used NASG in the management of PPH.

The magnitude of the utilization of NASG among obstetric care providers in the current study is comparable with the study done in the Maharashtra state of India, 35% [26], and the Ogun state of Nigeria, 36% [22]. The current study's finding is lower than those of studies among obstetric care providers conducted in Ibadan, Nigeria, 74% [27], and northern Ethiopian Tigray region, which had a rate of 64.2% [25], compared with the study done in Jimma, 36.2% [18], and study done in Addis Ababa, 39.3% [20]. This variation might be brought down due to the difference in the study setting, study participants, sample size, and availability of NASG. The other reason for the variation of NASG utilization might be due to country-to-country differences in scaling up and awareness creation among healthcare providers. In addition, the reason for the most significant difference between the current study and the study done in the Tigray region could be that the introduction of the instrument NASG in Ethiopia started in the northern part of the country in 2011. On the other hand, it was greater compared to the result that 14.1% of respondents used a nonpneumatic antishock garment to control postpartum hemorrhage in Ondo state, Nigeria [16]. The possible reason for the difference could be due to differences in the study setting, larger number of study participants, and larger number of public hospitals included in the current study.

This study indicated that obstetric care providers working in tertiary hospitals utilize NASG 35% less likely than those working in primary hospitals, and this could be because primary hospitals usually make referrals to tertiary and general hospitals for definitive management due to limited resources both material and human to manage a postpartum hemorrhage, so that the patients can be transported safely to a higher level of healthcare.

Findings from this study revealed that training on NASG was significantly associated with the utilization of NASG. Obstetric care providers who had training 4 times more utilized NASG than those who have no training. This finding agrees well with previous studies conducted in Addis Ababa [20]. This could be because attending training can help healthcare providers gain more understanding about how to apply and remove nonpneumatic antishock garments, enabling them to better utilize them.

This study result revealed statistical significance between providers' utilization of NASG and the existence of protocol; those who have protocol utilized it 2.7 more likely than those who did not have it. This is consistent with studies done in Ondo state of Nigeria [16] and done in Addis Ababa [20].

The protocol guides providers in how, when, and to which patient the NASG should be applied to; it also helps providers to build confidence and improve their readiness in utilizing NASG for PPH management. Contrary to this finding, a study done in selected southern zones in Arba Minch [21] reported no significant statistical relationship between the existence of NASG protocol and professionals' utilization of NASG. The possible reason for this could be due to differences in the study setting.

Another factor that was statically linked with the use of nonpneumatic antishock garments by healthcare workers in this study was the availability of nonpneumatic antishock garments.

Those who have nonpneumatic antishock garments in the facility are 4.6 times more likely to use them than those who have no nonpneumatic antishock garments in the facility. This agrees with studies done in Ondo state of Nigeria [16].

The results of the current study show that knowledge of NASG and its use is strongly correlated. People with good knowledge are around 2.5 times more likely to use nonpneumatic antishock garments than those with poor knowledge. Studies from the Edu state of Nigeria, northern Ethiopia, and Jimma, Ethiopia, support this [18, 25, 28].

This may be due to the fact that obstetric care providers will feel more confident applying the NASG if they have more knowledge. Contrary to the current study, findings from the Bayelsa state of Nigeria stated a nonsignificant association between professional's knowledge and utilization of NASG [24]. The discrepancy could be due to the different types of obstetric care providers included in the current study, whereas only midwives were included in the other study. The other reason might be the difference in sample size used and cross-country health policy differences.

In addition, a study conducted among midwives in Ogun state of Nigeria reported an inverse relationship between the midwives' knowledge and their utilization of nonpneumatic antishock garment in the management of postpartum hemorrhage [22]. This could be a result of the fact that the midwives have a good knowledge of NASG, but there was no NASG to be utilized in the management of postpartum hemorrhage.

Likewise, respondents' attitudes were significantly associated with their utilization of NASG for PPH management (*p* < 0.015). Compared with providers with a negative attitude towards the NASG, providers with a positive attitude were 2.4 times more likely to use the NASG. This finding is in line with studies conducted in the Ogun state of Nigeria and another 3 studies conducted in Ethiopia [18, 21, 22]. Contrary to this finding, a study from Ondo state, Nigeria [16], reported no relationship between attitude towards NASG and professionals' utilization of NASG. The possible reason for this could be due to methodological differences in computing attitude and items for assessing attitude and country-to-country differences.

## 5. Strength and Limitations of the Study

### 5.1. Strength of the Study

- The study was conducted at the regional level and incorporated different professionals, obstetricians, IEOS, general practitioners, nurses, and midwives which can help to make appropriate inferences

### 5.2. Limitations of the Study

- Recall bias was there because healthcare professionals were asked about their past utilization of NASG

## 6. Conclusion and Recommendation

However, the utilization of NASG can be greatly influenced by different factors, and we found that less than one-third of study participants have used the NASG in the management of PPH in Sidama regional state public hospitals, and this finding is low compared with other studies. Levels of hospitals, training about NASG, availability of NASG, availability of protocol, respondents' attitude, and knowledge are significantly associated factors in the current study.

Based on the findings of this study, the following recommendations are forwarded to the concerned bodies.

### 6.1. For Health Facilities

Health providers' in-service and ongoing professional development training on the use of nonpneumonic antishock clothing for the management of maternal hemorrhage has to be strengthened.

To make NASG available for healthcare providers, the hospital management needs to closely monitor its availability, location, and functionality.

### 6.2. For Health Professionals

They need to be motivated and interested in utilizing NASG accordingly to protocol whenever the need to utilize NASG rises. They also need to be familiar with the institution's policies regarding NASG utilization and PPH management.

### 6.3. For Researchers

Since this study included only obstetric care providers, further exploratory studies including program planners and hospital managers need to explore barriers that affect providers' utilization of NASG.

### 6.4. For Nongovernmental Organizations

Stakeholders and local nongovernmental organizations that are working on improving maternal health should use this opportunity to increase the accessibility of NASG and build the capacity of obstetric care providers.

## Figures and Tables

**Table 1:** Sociodemographic characteristics of obstetric care providers who participated in the study and worked in public hospitals of Sidama region, Ethiopia, 2022 (*N* = 394).

Variables	Category	Frequency	Percentage
Age in years	20-25	94	24.1
	25-29	159	40.4
	30-35	125	31.7
	Above 35	16	3.8

Sex	Female	162	41.1
	Male	232	58.9

Religion	Protestant	219	55.6
	Orthodox	137	34.8
	Muslim	20	5.1
	Catholic	13	3.3
	Other^∗^	5	1.3

Marital status	Single	110	28.2
	Married	279	70.5
	Others^∗∗^	5	1.3

Educational level	Diploma	83	21.1
	BSc	281	71.3
	Masters and above	30	7.6

Professional	Midwives	346	87.8
	Nurses	15	3.8
	GP	16	4.1
	IEOS	9	2.3
	Obstetrician	8	2.0

Job experience	1-5	273	69.3
	6-10	104	26.4
	>10	17	4.3

^∗^Other = Apostolic, Jehovah's Witness. ^∗∗^Others = divorced, married but living apart.

**Table 2:** Facility-related characteristics of obstetric care providers who participated in the study and worked in public hospitals of Sidama region, Ethiopia, 2022 (*N* = 394).

Variables	Category	Frequency	Percentage
Level of the hospital (*N* = 394)	Primary	161	40.9
	General	113	28.7
	Tertiary	120	30.5

Trained about NASG (*N* = 394)	Yes	86	21.8
	No	308	78.2

Availability of NASG in a facility (*N* = 394)	Yes	214	54.3
	No	117	29.7
	I do not know	63	16.0

Number of NASG (*N* = 214)	One	132	61.7
	More than one	38	17.8
	I do not know	44	120.6

Know the place of NASG (*N* = 214)	Yes	138	64.5
	No	76	33.5

Place of NASG after usage (*N* = 138)	Labor ward	105	76.1
	Not labor ward	33	23.9

Organization providing fund (*N* = 394)	Yes	78	19.8
	No	137	34.8
	I do not know	179	45.4

Protocols for NASG management (*N* = 394)	Yes	148	36.5
	No	190	49.2
	I do not know	56	14.2

Have wall chart displayed (*N* = 394)	Yes	172	42.9
	No	201	51.8
	I do not know	21	5.3

Working unit/ward^∗^	Labor and delivery	277	70.7
	ANC	51	13.0
	FP	12	3.1
	PNC	32	8.2
	Post OP (CS)/OR	50	12.8
	Gyn OPD	23	5.9

Have experienced staff	Yes	114	28.9
	No	165	41.9
	I do not know	115	29.2

Is NASG part of the PPH protocol before referral?	Yes	150	38.1
	No	213	54.1
	I do not know	31	7.8

^∗^Multiple responses.

**Table 3:** Knowledge of respondents who participated in the study and were working in public hospitals of Sidama region, Ethiopia, 2022 (*N* = 394).

Variable	Category	Frequency	Percent
Seen the NASG	Yes	300	76.1
	No	94	23.9

NASG looks like (*N* = 300)	The bottom half of a suit	245	81.7
	A gown	21	7.0
	A trouser	29	9.7
	I do not know	5	1.7

Knew NASG as it is used for preventing complications from PPH	Yes	378	95.9
	No	16	4.1

NASG is made from^#^	Velcro^∗^	115	29.3
	Neoprene^∗^	102	26.0
	I do not know	273	69.6

Number of segments of NASG	Nine	105	26.6
	Six	216	54.8
	Five	17	4.3
	Four	15	3.8
	I do not know	41	10.6

Starting segment while applying NASG	Lower or ankle^∗^	262	66.5
	Abdominal	88	22.3
	I do not know	44	11.2

Starting segment while removing NASG	Lower or ankle^∗^	244	61.9
	Abdominal	99	24.9
	I do not know	52	13.2

Segment adjusted when a woman experiences difficulty breathing with the NASG	Abdominal^∗^	261	66.2
	Thigh	60	15.2
	Leg	36	9.1
	I do not know	37	9.4

NASG applied to women having PPH when^#^	*Bleeding* > 750 *ml*^∗^	434	115.1
	*Systolic* *BP* > 90 *mm*^∗^	162	43.0
	*Pulse* < 110 *bpm*^∗^	140	37.0
	I do not know	139	36.9
	After stabilizing for 2 or more hours^∗^	4	1.9
	When the hg is 7 g/dl or more and hematocrit of about 20%^∗^	120	57.1
	Pulse rate less than 100 bpm^∗^	130	61.9
	Diastolic bp of 90 mmhg or more^∗^	97	46.2
	When the woman is awake/stable^∗^	136	64.8
	*Bleeding* < 50 *ml*/*hr*^∗^	93	44.3
	I do not know	32	15.2

Contraindication to the use of NASG^#^	Viable fetus^∗^	253	72.9
	Dyspnea^∗^	160	46.3
	Mitral stenosis^∗^	93	26.8
	Congestive heart failure^∗^	86	20.6
	Pulmonary hypertension^∗^	71	25.1
	Bleeding above the level of the diaphragm^∗^	98	27.7
	I do not know	108	31.6

Procedure can be performed on women on NASG^#^	IV line^∗^	288	74.0
	Vaginal surgery^∗^	157	40.4
	Abdominal surgery^∗^	109	28.0
	Transport to other facilities^∗^	184	47.3
	I do not know	64	26.5

Types of knowledge			

Good knowledge		220	55.8%

Poor knowledge		174	44.2%

^#^Multiple responses. ^∗^Correct response.

**Table 4:** Attitude of respondents who participated in the study and were working in public hospitals of Sidama region, Ethiopia, 2022 (*N* = 394).

Variables	Disagree	Neutral	Agree
Every healthcare personnel should know about the use of the nonpneumatic antishock garment	186 (47.2%)	11 (2.8%)	197 (50.0%)
The patient does not have to have the nonpneumatic antishock garment (NASG) applied upon	276 (70.1%)	19 (4.8%)	98 (25.1%)
Antishock garment (NASG) is only beneficial to people in rural areas/primary care settings	195 (49.5%)	28 (7.1%)	171 (43.4%)
The garment (NASG) is only meant to be applied by doctors	199 (50.5%)	27 (6.9%)	168 (42.6%)
The garment should be a must in every healthcare facility that has maternity service	205 (52.0%)	19 (4.8%)	170 (43.1%)
NASG is safe and effective in preventing complications of PPH	155 (39.3%)	103 (26.1%)	136 (34.6%)
I am motivated to use NASG during PPH because of its effectiveness	73 (18.1%)	48 (12.4%)	273 (69.4%)
There is no need for NASG since it is not readily available	221 (56.1%)	29 (7.5%)	144 (36.5%)
NASG saves time, energy, and life	50 (12.7%)	146 (36.5%)	198 (50.8%)
The garment is expensive, therefore not affordable	162 (41.1%)	51 (12.9%)	181 (45.9%)
*Attitude category*			
Positive	228 (57.9%)		
Negative	166 (42.1%)		

**Table 5:** Utilization of nonpneumatic antishock garment by respondents who participated in the study and were working in public hospitals of Sidama region, Ethiopia, 2022.

Variables	Category	Frequency	Percent
The last time an antishock garment was used (*N* = 121)	Less than 1 month ago	27	22.3
	Between 1 month and 3 months	7	5.8
	Between 3 months and 6 months	18	14.9
	More than 6 months ago	46	38.0
	I do not remember it	23	19.0

Reason for nonutilization^#^	Availability of another method	160	58.0
	Lack of training	229	82.6
	Not aware of the existence of NASG in the facility	105	38.0
	No patient needs it	57	20.7

Do you use NASG every time there is PPH (*N* = 121)	Yes	7	5.83
	No	114	94.16

If not, when do you use it^#^	When other methods fail	18	9.4
	When PPH with shock	84	44.2
	When active bleeding	71	37.3
	I do not remember	17	24.4

^**#**^Multiple responses are possible.

**Table 6:** Bivariable and multivariable logistic regression analyses for factors associated with the utilization of nonpneumatic antishock garments among obstetric care providers in public health facilities of Sidama region, Ethiopia, 2022.

Variables		Utilization of NASG		COR (95% CI)	AOR (95% CI)	*p* value
		No	Yes			
Gender	Female	124	38	1	1	
	Male	149	83	1.818 (1.157, 2.856)^∗^	1.402 (0.738, 2.664)	1.020

Educational level	Diploma	60	23	1		
	Bachelor	200	82	1.12 (0.64, 1.94)	0.694 (0.321, 1.50)	0.353
	Masters and above	13	17	3.41 (1.43, 8.12)^∗^	1.331 (0.382, 0.382)	0.650

Job experience	1-5	196	77	1	1	
	6-9	68	36	1.78 (1.108, 2.87)	1.581 (0.790, 3.161)	0.196
	≥10	9	8	1.92 (0.704, 2.23)	1.05 (0.272, 4.055)	0.606

Level of hospital	Primary	93	68	1	1	
	General	73	40	6.012 (3.126, 11.587)^∗^	0.779 (0.412, 1.548)	0.506
	Tertiary	107	13	4.51 (2.256, 9.017)^∗^	0.355 (0.132, 0.952)	0.04^∗∗^

Training NASG	No	244	64	1	1	
	Yes	29	57	7.494 (4.433, 12.66)^∗^	4.183 (2.167, 8.075)	0.000^∗∗^

Availability NASG	No	111	6	1	1	
	Yes	106	108	18.85 (7.94, 44.73)^∗^	4.603 (1.603, 13.24)	0.005^∗∗^
	I do not know	56	7	2.312 (0.742, 7.27)	2.593 (0.718, 9.364)	

Protocol about NASG	No	160	30	1		
	Yes	66	82	6.62 (3.99, 11.00)^∗^	2.758 (1.269, 5.996)	0.010^∗∗^
	I do not know	47	9	1.021 (0.453, 2.30)	1.562 (0.539, 4.521)	0.625

Wall charts	No	165	36	1		
	Yes	91	81	3.986 (2.494, 6.369)^∗^	1.012 (0.475, 2.157)	0.957
	I do not know	17	4	1.432 (0.493, 4.163)	0.924 (0.206, 4.140)	0.917

Have experienced staff	No	137	28	1	1	
	Yes	40	74	9.05 (5.17, 15.84)^∗^	2.051 (0.945, 4.45)	0.069
	I do not know	98	19	0.968 (0.511, 1.83)	1.067 (0.445, 2.554)	0.885

Knowledge	Poor	151	23	1	1	
	Good	122	98	5.274 (3.157, 8.808)^∗^	2.506 (1. 26, 4.984)	0.009^∗∗^

Attitude	Negative	136	30	1	1	
	Positive	137	91	3.011 (1.871, 4.847)^∗^	2.381 (1.189, 4.766)	0.015^∗∗^

Note: ^∗^statistically significant at *p* value < 0.25. ^∗∗^*p* < 0.05, 95% CI. 1 = reference group.

## Data Availability

Although the researcher cannot submit data to a repository due to prior agreement with the study participants, the datasets used and/or analyzed during the current study are available from the corresponding author on reasonable request.

## Abbreviations

AOR: Adjusted odds ratio
CI: Confidence interval
COR: Crude odds ratio
CHAI: Clinton Health Action Initiative
EDHS: Ethiopian Demographic Health Survey
NASG: Nonpneumatic antishock garment
MMR: Maternal mortality rate
OH: Obstetric hemorrhage
PPH: Postpartum hemorrhage.
